# Limited cross-protection induced by live attenuated ASF vaccines emphasizes caution for field use

**DOI:** 10.1080/22221751.2026.2741785

**Published:** 2026-09-28

**Authors:** Anna Lacasta, Christopher L. Netherton, Xinxin Niu, Dongming Zhao, Linda K. Dixon, Ana Luisa Reis

**Affiliations:** a International Livestock Research Institute, Nairobi, Kenya; b Vaccine and Infectious Disease Organization, Saskatoon, Canada; c ThePirbright Institute, Surrey, UK; d State Key Laboratory of Animal Disease Control and Prevention, Professional Laboratory for African Swine Fever (Harbin), National High Containment Facilities for Animal Diseases Control and Prevention, Harbin Veterinary Research Institute, Chinese Academy of Agricultural Sciences, Harbin, People’s Republic of China

African swine fever virus (ASFV) causes an often-fatal disease of domestic pigs and wild boars with a high socio-economic impact. The complexity of the large DNA genome and virion structure have hindered vaccine development. Gene-deleted modified live attenuated vaccines (LAVs) have proven to be the most successful and offer great potential to support control measures. However, their use introduces some safety risks, including transmission of the vaccine, potential genome mutations or recombination with field strains resulting in increased virulence. For successful vaccination, it is critically important to establish if the vaccine will protect against circulating strains to ensure efficacy and reduce opportunities to generate hybrid recombinants between the vaccine and field strains. In this commentary, we address the implications of recent studies demonstrating limited cross-protection offered by genotype II ASF LAVs against other circulating heterologous ASFV strains. These findings limit the safe and effective use of genotype II LAVs in these regions. We discuss priorities for future research to improve ASF LAVs and important considerations for their use in the field.

## ASFV distribution and diversity

ASFV originally spread to domestic pigs from a sylvatic cycle in East Africa and has been detected in most sub-Saharan countries. The complex DNA virus genome encodes more than 190 proteins. Partial sequencing of the *B646L* gene identified at least 23 genotypes in Africa, of which genotypes I, II, VIII, IX and X have had an extended presence in pigs [1]. Genome sequencing classified these genotypes into three main clades, with shared ancestry (Figure 1 – Clades A, B, C). Comparative analysis of the combined protein sequences encoded by complete ASFV genomes from eleven B646L genotypes defined seven biotypes. This protein-based classification has been used to predict the potential cross-protective efficacy of ASFV vaccines (Figure 1) [2].

**Figure 1.**
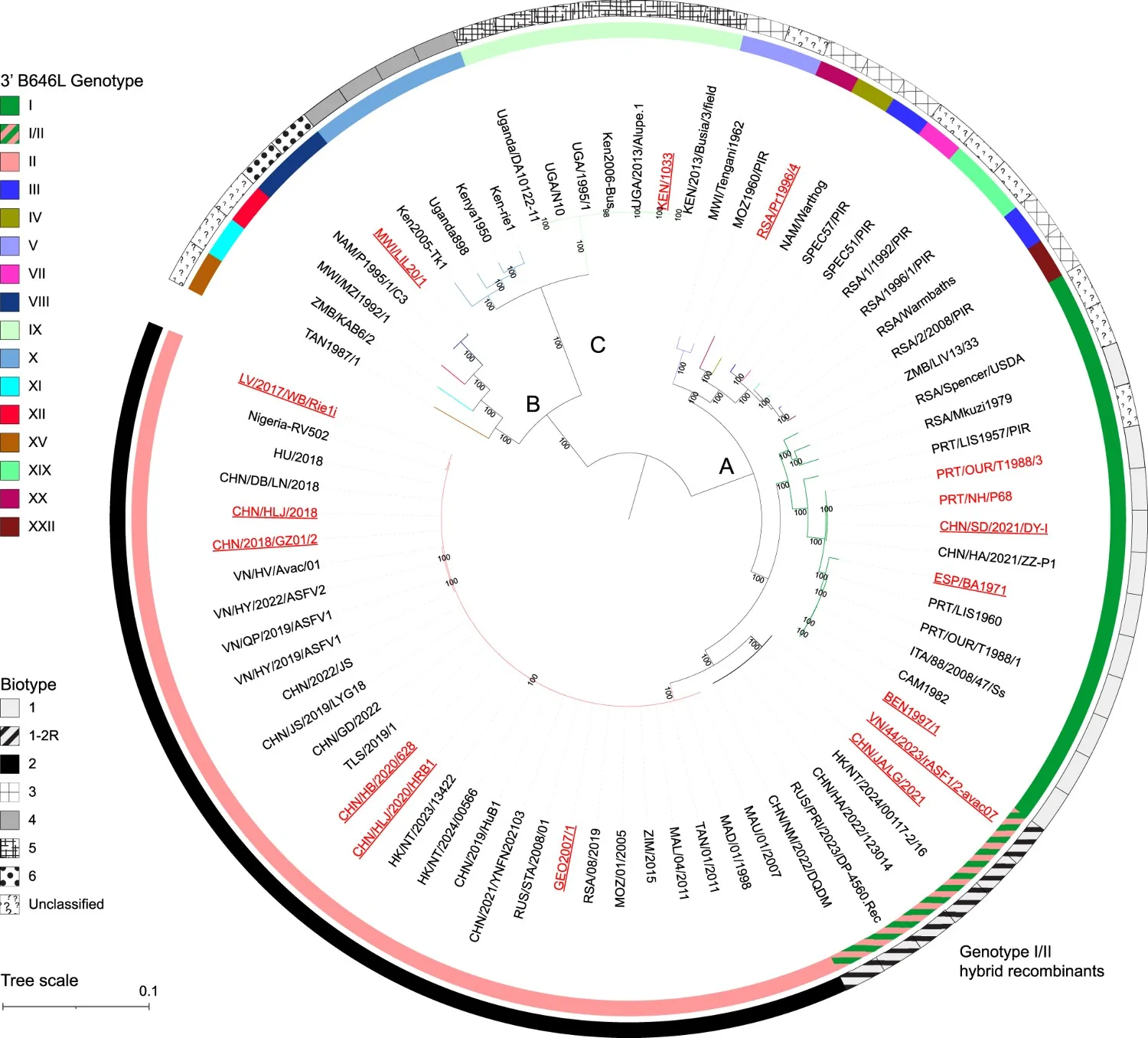
Genetic relatedness of genome sequences of African swine fever virus field isolates. The tree was inferred using the maximum likelihood method with a GTR + F + I + R4 model derived by IQ-TREE 3 and ModelFinder. The tree was rooted at the mid-point and the percentage of replicate trees in which the associated taxa clustered together in the ultrafast bootstrap test (10,000 replicates) in at least 95% of the trees are shown next to the branches. The 3’ B646L genotype and biotype for each sequence is coloured as per the keys, the three main clades are labelled A, B and C, and the genotype I/II recombinants are highlighted. Isolates discussed in the text are written in red text.

Genotype I (GI) ASFV spread to Europe in 1957 and again in 1960 and from there to the Caribbean and South America. GI was eventually eradicated from these countries in the 1990s, apart from Sardinia. Since 2007, genotype II (GII) ASFV has spread widely from Georgia across Europe, Asia and to parts of the Caribbean and Oceania [1]. In 2021, an attenuated GI strain, similar to strains previously reported in Portugal, was first detected in pigs in China [3]. The first recombinant between GI and GII (GI/II) hybrid was isolated in 2021 and contains 10 genomic fragments from each genotype interspersed across the genome. Afterwards, fully virulent GI/II hybrids were subsequently reported in China, [4], followed by Russia [5] and Vietnam, where by 2025 they represented up to 48% of the circulating isolates [6–8]. Further spread of GI/II hybrids in Asia seems inevitable. Diverse hybrid viruses generated between attenuated GI (PRT/OUR/T1988/3) and an attenuated gene-deleted GII (AQSΔB119L) were isolated after co-infections in cell cultures and in experimentally inoculated pigs. These GI/II hybrids caused acute ASFV in experimentally inoculated pigs, demonstrating rescue of virulent hybrids from the attenuated inoculated viruses [9]. Twelve complete genome sequences from GI/II hybrids generated experimentally *in vivo* showed marked diversity. Recombination sites were most frequently identified at flanking regions of ORFs related to virus attenuation [9]. The capacity for other ASFV genotypes circulating in Africa to recombine is unknown, although the probability may be significant since different genotypes have circulated simultaneously in the same areas for decades, and ASFV genome sequences are mostly well conserved. If progeny hybrid viruses gained functions that favour virus replication and spread, for example the ability to evade host innate or adaptive immune responses, then this could have resulted in apparent hot spots for recombination. Sequence analysis of individual genes *EP402R* and *EP153R* identified five recombinants in each gene, from seventy ASFV strains from Europe, Asia and Africa. The authors speculate that evasion of antibody and cellular immunity may drive positive selection of recombination in these genes [10]. The limited number of complete genome sequences of inter-genotype hybrid viruses available limits the current capacity to identify recombination hot spots more broadly across the genome.

## ASF LAVs: current status

Guidelines for testing of ASF LAVs include a comprehensive range of *in vivo* assays, from establishing the safety and efficacy across dose ranges, to overdose and repeat doses. The onset and duration of immunity, shedding and persistence of vaccine, and safety in pregnant sows and different aged pigs must also be established [11,12]. Several LAVs have been tested experimentally (reviewed in [8,11]), but only three GII LAVs have been licensed for use in Vietnam (ASFV-G-ΔI177L, ASFV-G-ΔMGF, ASFV-G-ΔI177L/ΔLVR). Vaccine-like field strains, associated with increased clinical signs and chronic disease, have been detected in Vietnam and Thailand. These findings indicate potential safety issues with the current licensed vaccines and the importance of developing LAVs with improved safety in the field [8,13,14].

## Limited cross-protective capacity of ASF LAVs

GII derived LAVs are effective against viruses related to GII Georgia 2007/1, but recent evidence shows limited cross-protective capacity against other genotypes and the GI/II hybrid strain. In Asia, the main concern is co-circulation of GI/II hybrids with GII viruses. Of the three GII LAVs tested, HLJ/18-7GD and ASFVG-Δ-MGF failed to protect any of the vaccinated pigs against GI/II challenge; and ASFV-G-ΔI177L induced protection against death in 2 of 4 pigs, although all vaccinated pigs showed clinical signs [4,15] (Table 1). A GI/II hybrid LAV, with the same seven deleted genes as those from the HLJ/18-7GD vaccine strain, failed to protect pigs from challenge with either the GI/II hybrid or GII strain [4]. Thus, safe use of these GII vaccines must be restricted to specific disease outbreaks or regions where GII strains alone are circulating to date. Hence, surveillance efforts are key for successful vaccine implementation. Ongoing research is directed at developing gene-deleted LAVs based on the GI/II hybrid; however, the selection of genes for deletion requires further optimization [16].

**Table 1.** Cross-protection induced by ASF genotype I and II LAVs.

(a) Vaccine (Genotype/Biotype)	(b) Prime Dose Log_10_	(c) Boost Dose Log_10_	(d) Challenge (Genotype/Biotype/Clade)	(e) Challenge Dose Log_10_	(f) Survival	(g) Signs	(h) Ref
ASFV-G-ΔI177L (II/2)	4	–	Georgia2010 (II/2/A)	2	5/5	1/5	[17]
ASFV-G-ΔI177L (II/2)	4	–	Ghana2021 (I/1/A)	2	4/5	4/5	[17]
ASFV-G-ΔI177L (II/2)	4	–	MWI/Lil20/1 (VIII/6/B)	2	0/5	5/5	[17]
ASFV-G-ΔI177L (II/2)	4	–	RSA/Pr1996/4 (XX/3/A)	4	0/5	5/5	[17]
ASFV-G-ΔI177L (II/2)	4	–	Kenya1033 (IX/5/C)	2	0/5	5/5	[17]
ASFV-G-ΔI177L (II/2)	4	–	Uganda1965 (X/4/C)	2	0/5	5/5	[17]
GΔDKE-Cmut (II/2)	4.7	4.7	Benin 1997/1 (I/1/A)	3	5/7	7/7	[19]
GΔDKE-Cmut (II/2)	4.7	4	Benin 1997/1 (I/1/A)	3	4/7	5/7	[19]
GΔDKE-Cmut (II/2)	4.7	4.7	Georgia 2007/1 (II/2/A)	3	7/7	1/7	[19]
GΔDKE-Cmut (II/2)	4.7	4.7	Kenya 1033 (IX/5/C)	2	0/6	6/6	[19]
Lv17/WB/Rie1 (II/2)	3*	3*	Arm2007 (GII/2/A) + ^$^Ken2006.Bus (IX/5/C)	1 + (in contact ^$^)	3/8	8/8	[18]
HLJ/18-7GD (II/2)	6	–	CHN/JA/LG/2021(I/II/1-2R/A)	3	0/5	0/5	[4]
HLJ/18-7GD (II/2)	6	–	CHN/HLJ/2018 (II/2/A)	3	5/5	5/5	[4]
HLJ/18-7GD (II/2)	6	–	CHN/HB/2020/628 (II/2/A)	1.5	5/5	2/5	[20]
HLJ/18-7GD (II/2)	6	–	CHN/HLJ/2020/HRB1 (II/2/A)	6	5/5	0/5	[20]
HLJ/18-7GD (II/2)	6	–	CHN/SD/2021/DY-I (I/1/A)	6	4/5	5/5	[20]
ASFV-G-ΔI177L (II/2)	4	–	VN/2023/rASFV/TN1 (I/II/1-2R/A)	3	2/4	4/4	[15]
ASFV-G-ΔMGF (II/2)	4	–	VN/2023/rASFV/TN1 (I/II/1-2R/A)	3	0/6	6/6	[15]
ASFVHNΔCD2vΔMGF (I/II /1-2R)	5	5	ASFV-HN (I/II/1-2R/A)	2	0/5	5/5	[16]
ASFVHNΔCD2vΔMGF (I/II /1-2R)	5	5	CHN/2018/GZ01/2 (II/2/A)	2	0/5	5/5	[16]
ASFVGZDECM3 (II/2)	5	5	CHN/2018/GZ01/2 (II/2/A)	2	5/5	0/5	[16]
BA71ΔCD2v (I/1)	6*	–	Georgia 2007/1 (II/2/A)	5*	5/5	0/5	[22]
BA71ΔCD2v (I/1)	4.5	–	Ken2006.Bus (IX/5/C)	2	1/6	6/6	[22]
BA71ΔCD2v (I/1)	6	–	Ken2006.Bus (IX/5/C)	2	1/6	6/6	[22]
BA71ΔCD2v (I/1)	4.5	4.5	Ken2006.Bus (IX/5/C)	2	3/6	3/5	[22]
BA71ΔCD2v (I/1)	6	-	ESP/BA1971 (I/1/A) + Ken2006.Bus (IX/5/C)	3 + 2	5/5	1/5	[22]

Column (a), shows the LAV nomenclature followed in brackets by the genotype and biotype designated numbers separated by forward slashes. For example (II/2), (I/1), (I/II/1-2R), (IX/5), (X/4), (VIII/6). The GI/II hybrid biotype is designated 1-2R [2]. Column (b), shows the LAV dose and (c) the boost dose as TCID_50_ or HAD_50_ log_10_. A hash sign indicates a boost was not applied. Column (d), shows the challenge virus nomenclature followed in brackets by the genotype, biotype and clade designated numbers separated by a forward slash. In two examples, a second challenge virus was delivered, and this is labelled with the designated name followed by genotype and biotype numbers in brackets. One of these is labelled Arm2007 (GII/2/A) + ^$^Ken2006.Bus (IX/5/C), and the second is labelled ESP/BA1971 (I/1/A) + Ken2006.Bus (IX/5/C). The route of delivery was by the intramuscular route unless labelled with an asterisk * to indicate the oral/nasal route or dollar sign ^$^ for challenge by direct contact. Column (e) shows the challenge virus dose as TCID_50_ or HAD_50_ log_10_. In the experiments which challenged first with ESP/BA1971 (I/1/A) followed by Ken2006.Bus (IX/5/C), different doses were used as indicated. Column (f) shows the number of pigs that survived challenge separated by a forward slash from the number of pigs that were challenged. Column (g) shows the numbers of pigs that showed clinical signs typical of ASFV compared to those challenged. These numbers are separated by a forward slash. Clinical signs recorded included fever, inappetence and lethargy. Column (h) gives the publication reference number.

In Africa, the diversity of circulating ASFV strains presents a greater challenge for LAVs, since in many countries several genotypes are circulating simultaneously [1]. Although GII has been detected in 9 African countries, additional genotypes are usually circulating [1]. Recent results showed that GII LAVs (ASFV-G-ΔI177L, GΔDKE-Cmut, ASFV-G-ΔMGF, Lv17/WB/Rie1, HLJ/18-7GD) induced strong protection against GII virus and three of them induced variable protection against GI, but no protection against the more divergent GIX [17–20] (Table 1). The ASFV-G-ΔI177L also failed to protect against virulent viruses from three additional genotypes (VIII, XX and X) [17] (Table 1), thus the potential use of GII LAVs in Africa is restricted. An attenuated GI LAV (BA71ΔCD2v) induced 100% cross-protection against GII challenge confirming the potential for cross-protection between GI and GII [21,22]. Similarly, low virulent GI field isolates PRT/NH/P68 and PRT/OUR/T1988/3 confer protection against homologous challenge, but only partial protection against GII [23,24]. This highlights the need for a more systematic approach to defining protection and cross-protection. It is unclear why BA71ΔCD2v induces robust cross-protection against genotype-II ASFV, but GII LAVs produce poorer cross-protection against GI ASFV. Importantly, the GI ESP/BA1971 and GII Georgia 2007/1 backbones are not directly comparable, as they differ in the copy number of several multigene families the functions of which are unknown and have variable sequences of many other genes. These examples underscore the importance of incorporating protein structure and function into future analyses of sequence divergence and the prediction of cross-protection.

## Predicting cross-protection

In principle, prediction of cross-protective ability should be possible from the *in silico* analysis of the outbreak strains to identify differences in the sequence of protective epitopes compared to the vaccine, which could reduce its effectiveness. Cellular immunity is considered essential for protection induced by LAVs [25–27] (for review see [28]) and some progress has been made in identifying antigens encoding protective T-cell epitopes, as demonstrated by the delivery of these in viral vectors resulting in partial protection against homologous virulent virus challenge [27, 28]. However, knowledge of protective epitopes is relatively limited and detailed transcription and translation data is only available for GII ASFV [29,30], with some proteomic data for genotype IX [31]. In addition, LAV models need to be developed to enable robust comparison of protective antigens and epitopes between different genotypes. Characterization of antigen-specific T-cell subsets correlating with protection [26,32] should help to refine the ability to predict virus T-cell epitopes with cross-protective potential. Methodological standardization between laboratories would allow integration of immunological datasets and better support application of machine learning techniques, that have been successfully used in the medical field [33] to veterinary data.

Although antibodies are known to play a role in protection, the mechanism is poorly understood. Virus neutralization by sera from vaccinated animals is only partially or not at all effective in cell culture. This precludes the potential use of this straightforward *ex vivo* assay to aid prediction of cross-protection. ASFV proteins that induce antibodies with potential roles in protection include proteins p30 (*CP204L*), p54 (*E183L*), and p72 (*B646L*). Immunization of pigs with these recombinant proteins induced antibodies that partially neutralized virus infection *in vitro* and led to a slight delay in the onset of clinical signs, however all immunized pigs eventually developed acute disease, high viraemia, and died at the same time as the controls [34].

The ASFV proteins pCD2v (*EP402R*) and C-type lectin pEP153R (*EP153R*) are required for haemadsorption of red blood cells to infected cells (HAD). The ability of sera from pigs immunized with selected attenuated ASFV strains to inhibit HAD to cells infected with diverse virulent ASFV strains enabled eight ASFV serotypes to be identified. Within serogroups, HAD inhibition correlated with the ability to cross-protect pigs against virulent virus challenge [35]. However, recombinant viruses from which *EP402R* and/or *EP153R* genes are deleted can induce good levels of protection against homologous challenge. This demonstrates that immune responses to these proteins are not essential for protection (for review see [11]). Although progress has been made towards prediction of cross-protection, a deeper knowledge of protective antigens and the immunological mechanism of protection is required. This information would accelerate the development of subunit vaccines.

Many other factors influence the protection and cross-protection induced by LAVs. One key determinant is the ability of the LAV to induce a robust and effective immune response. Equally important is the capacity of the challenge virus to evade or counteract this immunity. Further research is required to understand how variation in the gene complement or sequences of ASFV proteins modulate host immune responses.

## Research needs: improving LAV safety and other vaccine approaches

Research is also needed to provide LAVs with improved safety profiles by limiting levels of replication, persistence, and spread from animals, while maintaining high levels of protection. Reducing vaccine persistence and shedding is important to limit opportunities for the vaccine to disperse in the environment, leading to the possibility of reverting and becoming virulent or recombining with circulating strains. Recombination may result in the generation of hybrid strains that are resistant to immunity induced by the vaccine. Achieving these aims will require better knowledge of the virus interactions with the host, particularly those involved in pathogenesis and induction of protective immune responses.

A further urgent need is to develop LAVs with serological capacity to differentiate infected from vaccinated animals (DIVA), to facilitate post-vaccination monitoring in the field. Deletion of non-essential genes encoding immunogenic proteins can produce DIVA vaccine candidates which do not induce antibody responses against these proteins, whereas infections with field strains do induce responses. Evaluation of vaccine candidates with the *EP153R* gene, coding for pEP153R [36] or *EP402R* gene, coding for pCD2v, deleted [37], combined with a positive assay for proteins expressed by vaccine and field strains, has provided promising DIVA results, although further field testing is required. Developing alternative vaccine approaches, including subunit vaccines, could provide a safer and flexible approach to produce cross-protective vaccines. However, subunit vaccines have not yet demonstrated sufficient efficacy for use.

## Priorities for vaccine development

Currently only GII LAVs have been tested and licensed. These have the potential to be used in countries where only GII is circulating, including Europe and countries in Asia where the GI/GII hybrid has not yet been detected. In other countries, limited use of GII LAVs in selected regions may be considered, but this requires good knowledge of the distribution of circulating ASFV strains. There is an urgent need for additional vaccines that protect against area-specific circulating strains.

A declaration from WOAH, FAO and the African Union agreed on considerations for use of ASF vaccines and future activities in Africa [38]. Further development of GIX LAVs for use in East Africa was encouraged, although co-circulation of other genotypes would complicate the deployment of vaccines. Moreover, issues associated with the deployment in rural and smallholder settings would also need to be addressed. Practical barriers include the requirement for increased genome surveillance in rural Africa to establish the potential for use of genotype-matched LAVs and the need for cold chains, which is a must for vaccine distribution in those areas.

It is crucial that the relevant veterinary authorities carefully consider the guidelines from international organizations (WOAH and FAO) before vaccination [12,39,40]. Considerations include whether the country is disease free, disease is broadly endemic or present in specific regions, and the desired outcome, for example, reduction or elimination of disease. The availability of infrastructure for vaccine deployment and post-vaccination monitoring, the cost and effectiveness of the vaccination campaign, should also be considered. Reduction in disease may be the accepted outcome for countries in which disease is endemic and live pigs or pork products are not exported. Disease eradication is likely to be the aim for exporting countries. In parallel, continued implementation of awareness campaigns and biosecurity is essential [12,39,40]. Considerable investment is required for vaccine development to achieve international standards of safety, efficacy, and manufacture at the scale needed for field use. Protecting the intellectual property generated through vaccine research, including through patents, helps support and incentivize this investment.
